# Incidence and predictors of radial artery occlusion following transradial coronary angiography: the proRadial trial

**DOI:** 10.1007/s00392-022-02094-z

**Published:** 2022-09-08

**Authors:** Julia Schlosser, Laura Herrmann, Tanja Böhme, Karlheinz Bürgelin, Nikolaus Löffelhardt, Thomas Nührenberg, Kambis Mashayekhi, Christian M. Valina, Franz-Josef Neumann, Willibald Hochholzer

**Affiliations:** 1 Department of Cardiology and Angiology II, University Heart Center Freiburg Bad Krozingen, Suedring 15, 79189 Bad Krozingen, Germany; 2 Department of Internal Medicine and Cardiology, Klinikum Wuerzburg Mitte, Würzburg, Germany

**Keywords:** Radial artery occlusion, Transradial angiography, Vascular complication, Predictor, Treatment of radial occlusion

## Abstract

**Objectives:**

This study investigated the contemporary incidence and predictors of radial artery occlusion as well as the effectiveness of antithrombotic treatment for radial artery occlusion following transradial coronary angiography.

**Background:**

The radial artery is the standard access for coronary angiography and even complex interventions. Postprocedural radial artery occlusion is still a common and significant complication.

**Methods:**

This prospective study enrolled 2004 patients following transradial coronary angiography. After sheath removal, hemostasis was obtained in a standardized fashion. Radial artery patency was evaluated by duplex ultrasonography in all patients. In case of occlusion, oral anticoagulation was recommended and patients were scheduled for a 30-day follow-up including Doppler ultrasonography.

**Results:**

A new-diagnosed radial occlusion was found in 4.6% of patients. The strongest independent predictors of radial occlusion were female sex and active smoking status. In the subgroup of patients with percutaneous coronary interventions, female sex followed by sheath size > 6 French were the strongest predictors of radial occlusion. 76 of 93 patients with radial occlusion received an oral anticoagulation for 30 days. However, reperfusion at 30 days was found in 32% of patients on oral anticoagulation.

**Conclusion:**

The incidence of radial artery occlusion following coronary angiography in contemporary practice appears with 4.6% to be lower as compared to previous cohorts. Female sex and smoking status are the strongest independent predictors of radial occlusion followed by procedural variables. The limited effectiveness of oral anticoagulation for treatment of radial artery occlusion suggests a primarily traumatic than thrombotic mechanism of this complication.

**Graphical abstract:**

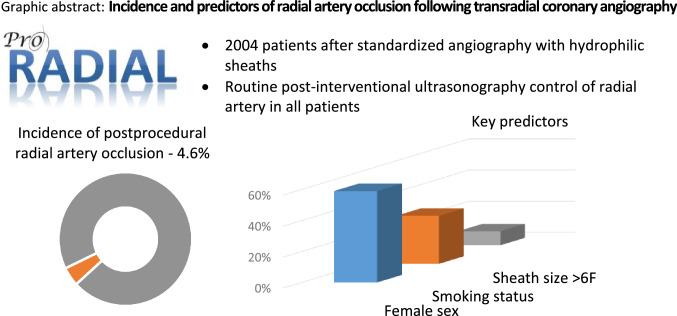

**Supplementary Information:**

The online version contains supplementary material available at 10.1007/s00392-022-02094-z.

## Introduction

The transradial access is recommended by current guidelines as a standard approach for diagnostic coronary angiography and interventions [1, 2]. Several large, randomized trials have demonstrated that this access is associated with a significant reduction in bleeding as well as lower morbidity and cardiac mortality compared to the femoral access [3–5]. More recently, a randomized trial showed that the radial approach might be even preferable for complex coronary interventions needing large-bore access [6]. In this study, the radial compared with femoral access was associated with a significant reduction in vascular access site bleeding and other clinically obvious vascular complications without affecting procedural success.

Despite many advantages, the radial access can be associated with certain disadvantages such as pain or vascular complications. The persistent occlusion of the radial artery is certainly the key complication of this vascular approach [7]. Since the majority of the patients remain asymptomatic due to collateral arterial perfusion, this complication is still underdiagnosed in clinical routine. However, silent radial occlusion is a serious obstacle for subsequent angiographies.

In early studies, the incidence of radial artery occlusion following angiography varied from less than 1% to up to 33% [8, 9]. More recent data indicate that the mean incidence is still above 5% [10]. Further analyses identified female sex, larger sheath size, and lower body mass index as potential risk factors for radial occlusion [8, 10, 11]. Other studies described different techniques such as a patent hemostasis protocol or prophylactic ipsilateral ulnar compression for prevention of radial occlusion [7, 12, 13]. The current incidence of radial artery occlusion might be even lower than previously reported, given the evolution of materials, techniques, and anticoagulatory strategies in recent years. However, the use of large-bore sheaths, and the use of radial access for time-consuming, complex coronary interventions might also have negatively affected this number. Thus, the present study sought to investigate the current incidence and predictors of radial artery occlusion following transradial coronary angiography in patients without or with percutaneous coronary intervention including complex procedures with large-bore access. The key focus was the evaluation of patient’s and procedural characteristics to allow identification of patient subgroups at high risk for radial occlusion post angiography. Another focus of this study was to evaluate the effectiveness of antithrombotic treatment and the reperfusion rate of radial artery in follow-up of 30 days. The treatment of radial occlusion is controversy and there is still no unique standard recommendation.

## Methods

### Study population

**Fig. 1:**
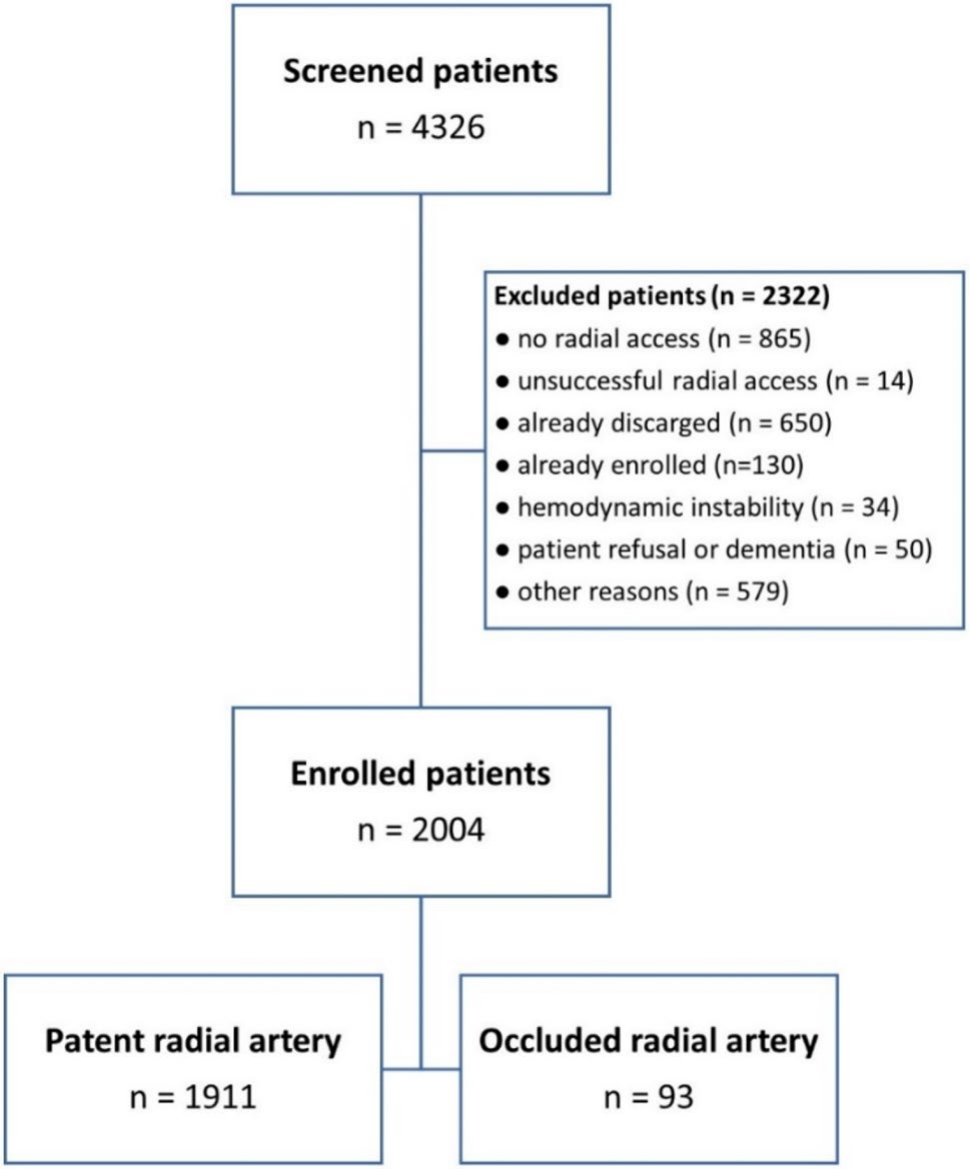
Flow diagram for study participants

The proRadial study was a prospective observational study enrolled patients undergoing coronary angiography with or without percutaneous coronary intervention (PCI) between March 2019 and August 2020 at the University Heart Center Freiburg · Bad Krozingen (Campus Bad Krozingen, Germany). All patients underwent coronary angiography were screened for this study. Key inclusion criteria were age ≥ 18 years and a successful transradial angiography. Patients not able to provide a valid written informed consent (e.g., due to dementia) or in a state of dependence to our hospital were not included. The main reasons for unsuccessful enrollment were early discharge of patients or transfer to another department for subsequent treatment (mainly cardiac surgery Fig. 1). The primary endpoint was radial artery occlusion at the vascular access site detected by vascular ultrasonography on day 1 following coronary angiography. Key secondary endpoints were bleeding complications at the vascular access site, defined as severe local hematoma (diameter > 5 cm) or relevant bleeding according to the BARC criteria (type ≥ 2) [14]. Other clinical symptoms such as pain or neurological impairment were evaluated. In case of radial occlusion, an oral anticoagulant was recommended regardless of symptoms for at least 30 days according to the local standard of care. The selection of treatment was left to the discretion of the treating physician including if a novel oral anticoagulant (NOAC) or vitamin K antagonist was used. A 30-day clinical follow-up including duplex ultrasonography was scheduled in all patients with radial artery occlusion. All participants provided written informed consent prior to enrollment and any study procedures. This study was approved by the ethics committee of the University of Freiburg (Germany) and complied with the principles of the Declaration of Helsinki. The study was registered at the German Clinical Trials Register (drks.de, identifier: DRKS00016962).

### Procedural details

Transradial coronary angiography was performed in a very standardized fashion. Only hydrophilic coated introducer sheaths from 5 to 7 French and 10 cm length were used (Glidesheath Slender^®^, Terumo, Eschborn, Germany). Following radial puncture, all patients received 5000 IU of heparin intravenous and 200 µg of nitroglycerine if not clinically contraindicated. In case of PCI, patients received 100 IU heparin per kg followed by adapted doses of heparin to achieve an activated clotting time of more than 250 s. For radial compression, all patients received the same radial compression device (TR BAND® Radial Compression Device, Terumo, Eschborn, Germany) according to manufacturer instructions and using patent hemostasis. After introducer sheath was withdrawn, the TR BAND^®^ compression device was applied proximal to the puncture site and fixed. In the absence of plethysmographic waveform, hemostatic compression pressure was decreased to the point where plethysmographic waveform returned and hemostasis was maintained. The first check for patent hemostasis was already done directly in the catheterization laboratory. Thereafter, patients were under the supervision of a dedicated nursing team, which gradually reduced the pressure of the device under consecutive control of the local status and peripheral blood flow. The radial compression device could be removed after 4 h in almost all patients according to manufacturer instructions.

On the day following angiography, patients underwent a physical examination of the arm and the vascular access site. A duplex ultrasonography was performed in all patients using a portable device (Vscan Extend, Ultrasound GE Healthcare GmbH, Solingen, Germany). In case of absent flow of the radial artery, patients were sent for a further, more thorough ultrasound study to our department of angiology. Confirmation of radial artery occlusion was defined as an absence of antegrade flow determined by duplex ultrasonography.

### Statistics

Data are presented as median with interquartile range or numbers with percent. Continuous variables were compared using the Mann–Whitney *U* test, and categorical by Chi-squared test. All variables with a *p* value < 0.30 in direct group comparison were selected for binary logistic regression analyses. In addition, the following variables with a plausible potential pathophysiological relationship to radial occlusion were included in these analyses even if the p values were not < 0.30: previous transradial angiography, diabetes, duration of examination, sheath size, and number of catheter exchanges. Independent predictors for radial artery occlusion were identified by stepwise binary logistic regression analyses with backward elimination. The strength of association with radial artery occlusion in the single models was determined by Wald chi-square. *p* values < 0.05 were considered as statistically significant. IBM SPSS Statistics, version 25 (IBM Corporation, New York, USA) was used for statistical analyses.

## Results

### Patient characteristics

In total, 4326 patients were screened for this study. Finally, 2004 patients fulfilling all inclusion and no exclusion criteria provided written informed consent (Fig. 1). A radial occlusion was found in 4.6% of cases (93/2004). The mean age of population was 70 years and 65.6% of the patients were male.

Table 1 shows the baseline characteristic of patients stratified into groups with or without radial artery occlusion. Patients with diagnosed radial artery occlusion were significantly younger as compared to patients without radial occlusion with a mean age of 66 vs. 70 years, almost twice as often female, as well as smaller, and had a lower body weight. Patients in the group with radial occlusion had less frequently a history of coronary heart disease or arterial hypertension, but had a higher thrombocyte count and were almost twice as often active smokers.

**Table 1:** Baseline characteristics

	Radial artery occlusion		*p* value
	No (*n = *1911)	Yes (*n = *93)	
Age (years)	70 [61–78]	66 (57–76)	0.02
Female	635 (33.2%)	55 (59.1%)	< 0.001
Body height (cm)	172 [165–178]	168 (163–175)	0.002
Body weight (kg)	80 [70–92]	75 [66–88]	0.01
Body mass index (kg/cm^2^)	27.4 [24.6–30.6]	26.3 [23.7–30.8]	0.12
Medical history			
Impaired LV function (EF < 55%)	471 (24.6%)	17 (18.3%)	0.16
Coronary artery disease	1357 (71.0%)	55 (59.1%)	0.01
Congestive heart failure	260 (13.6%)	18 (19.4%)	0.12
Previous myocardial infarction	446 (23.3%)	18 (19.4%)	0.37
Previous PCI	824 (43.1%)	36 (38.7%)	0.40
Previous transradial angiography	608 (31.8%)	28 (30.1%)	0.73
Previous CABG	74 (3.9%)	1 (1.1%)	0.17
Peripheral artery disease	146 (7.6%)	7 (7.5%)	0.97
Previous stroke or TIA	121 (6.3%)	4 (4.3%)	0.43
Cardiovascular risk factors			
Arterial hypertension	1502 (78.6%)	64 (68.8%)	0.03
Active smoking	305 (16.0%)	29 (31.2%)	< 0.001
Hypercholesterolemia	1434 (75.0%)	68 (73.1%)	0.68
Diabetes mellitus	498 (26.1%)	23 (24.7%)	0.78
Medication on admission			
Aspirin	1025 (53.6%)	52 (55.9%)	0.67
P2Y_12_-receptor inhibitor	381 (19.9%)	18 (19.4%)	0.89
Oral anticoagulant	361 (18.9%)	13 (14.0%)	0.24
β-Blockers	1020 (53.4%)	41 (44.1%)	0.08
Nitrates	46 (2.4%)	5 (5.4%)	0.08
Calcium channel blockers	424 (22.2%)	18 (19.4%)	0.52
Statins	990 (51.8%)	45 (48.4%)	0.52
Baseline laboratory results			
High sensitivity troponin T (ng/L)	13 [8–23]	11 [7–21]	0.12
Creatine kinase (U/L)	96 [69–141]	115 [81–173]	0.06
Creatinine clearance (mL/min)	74 [60–85]	74 [62–86]	0.87
C-reactive protein (mg/L)	2 [1–4]	2 [1–5]	0.20
LDL cholesterol (mg/dL)	106 [78–145]	104 [77–151]	0.62
Hemoglobin (g/dL)	12.3 [12.3–14.7]	11.7 [11.7–14.4]	0.10
Thrombocytes (× 10^3^/µL)	223 [189–262]	235 [200–297]	0.02
White blood cells (1/µL)	7060 [5930–8500]	7820 [5995–9190]	0.14

Data are presented as median [interquartile range] or number (percent). *p* values by either Mann–Whitney *U* or Chi-square test

*LV* left ventricle; *EF* ejection fraction; *PCI* percutaneous coronary intervention; *CABG* coronary artery bypass grafting; *TIA* transient ischemic attack

The peri- and postprocedural characteristics are shown in Table 2. Patients with radial artery occlusion underwent less frequently coronary intervention and had a trend towards multiple radial artery puncture attempts (4.3% vs. 1.8%). As expected, radial artery was not palpable in most of patients with radial artery occlusion. However, this was also the case in more than 4% of patients without radial occlusion. Many of patients with a diagnosed radial occlusion reported symptoms such as local pain in 37% or local paresthesia in 28%. Severe local hematoma (diameter > 5 cm) or BARC type 2 bleeding was found in 22% of patients with a radial artery occlusion as compared to 6% of patients without occlusion. Only one patient developed a pseudoaneurysm, which was treated conservatively. There were no patients with major neurological impairment or major bleeding (BARC type 3–5) at the vascular access site.

**Table 2:** Peri- and postprocedural characteristics

	Radial artery occlusion		*p* value
	No (*n = *1911)	Yes (*n = *93)	
Acute myocardial infarction	125 (6.5%)	3 (3.2%)	0.20
Urgent angiography	113 (5.9%)	4 (4.3%)	0.51
Multiple puncture attempts	34 (1.8%)	4 (4.3%)	0.08
PCI performed	767 (40.1%)	24 (25.8%)	0.006
Time of examination (min)	31 [18–58]	27 [18–50]	0.46
Radiation time (min)	5 [2–13]	4 [2–13]	0.48
Sheath size			0.79
5 F	65 (3.4%)	3 (3.2%)	
6 F	1717 (89.8%)	82 (88.2%)	
7 F	129 (6.8%)	8 (8.6%)	
Maximum catheter size			0.11
5 F	639 (33.4%)	40 (43.0%)	
6 F	1152 (60.3%)	46 (49.5%)	
7 F	120 (6.3%)	7 (7.5%)	
Number of catheter exchanges	1 [1, 2]	1 [0–2]	0.36
Vascular access site post-angiography			
Radial pulse non palpable	85 (4.4%)	92 (98.9%)	< 0.001
Local pain	238 (12.5%)	34 (36.6%)	< 0.001
Local paresthesia	279 (14.6%)	26 (28.0%)	< 0.001
Severe local hematoma (> 5 cm)	108 (5.7%)	20 (21.5%)	< 0.001

Data are presented as median [interquartile range] or number (percent). *p* values by either Mann–Whitney-*U* or Chi-square test

*PCI* percutaneous coronary intervention

### Predictors of radial artery occlusion

Several variables were significantly associated with radial artery occlusion in univariable analyses (Table 3). The strongest association with the primary endpoint was seen for female sex and active smoking followed by body height, PCI during the index angiography, and younger age. Multivariable regression analyses identified female sex and smoking status followed by levels of creatine kinase and PCI during the index angiography as independent predictors of radial artery occlusion (Table 4).

**Table 3:** Univariable binary logistic regression analyses for radial occlusion

	Odds ratio	95% CI	*p* value
Baseline characteristics			
Age (per year)	0.98	0.96–0.99	0.01
Female sex	2.91	1.90–4.45	< 0.001
Body height (per 1 cm)	0.97	0.94–0.99	0.004
Body weight (per 1 kg)	0.98	0.97–1.00	0.02
Body mass index (per 1 kg/cm^2^)	0.97	0.93–1.02	0.26
Impaired LV function (EF < 55%)	0.68	0.40–1.17	0.17
Coronary artery disease	0.59	0.39–0.90	0.02
Congestive heart failure	1.52	0.90–2.59	0.12
Previous transradial angiography	0.92	0.59–1.45	0.73
Previous CABG	0.27	0.04–1.96	0.20
Arterial hypertension	0.60	0.38–0.94	0.03
Active smoking	2.39	1.51–3.76	< 0.001
Diabetes	0.93	0.57–1.51	0.78
Oral anticoagulant	0.69	0.38–1.27	0.24
β-Blockers	0.72	0.48–1.09	0.13
Nitrates	2.30	0.89–5.94	0.08
Calcium channel blockers	0.84	0.50–1.42	0.52
High sensitivity troponin T (per 1 ng/L)	1.25	0.67–2.33	0.49
Creatine kinase (per 10U/L)	1.02	1.01–1.03	0.03
C-reactive protein (per 1 mg/L)	0.99	0.73–1.19	0.95
Hemoglobin (per 1 g/dL)	0.93	0.85–1.02	0.14
Thrombocytes (per 100 × 10^3^/µL)	1.22	0.99–1.50	0.07
White blood cells (per 1000/µL)	1.04	0.98–1.10	0.24
Peri- and postprocedural characteristics			
Acute myocardial infarction	0.47	0.15–1.53	0.21
Multiple puncture attempts	2.48	0.86–7.15	0.09
PCI performed	0.52	0.32–0.83	0.007
Time of examination (per min)	1.00	0.99–1.01	0.90
Sheath size > 6F	1.30	0.62–2.74	0.49
Maximum catheter size > 6F	1.22	0.55–2.68	0.63
Number of catheter exchanges	0.93	0.79–1.09	0.34

*CI* confidence interval; *LV* left ventricle; *EF* ejection fraction; *PCI* percutaneous coronary intervention; *CABG* coronary artery bypass grafting

**Table 4:** Multivariable binary logistic regression analyses for radial occlusion

	Wald *Χ*^2^	Odds ratio	95% CI	*p* value
Whole cohort (*n = *2004)				
Female sex	22.60	2.91	1.87–4.52	< 0.001
Active smoking	17.43	2.74	1.71–4.39	< 0.001
Creatine kinase (per 10U/L)	8.84	1.02	1.01–1.03	0.003
PCI performed	4.07	0.60	0.37–0.99	0.04
Cohort undergoing diagnostic angiography only (*n = *1213)				
Active smoking	17.79	3.21	1.90–5.53	< 0.001
Female sex	16.25	2.93	1.74–4.94	< 0.001
Time of examination (per min)	4.48	1.01	1.00–1.02	0.03
Cohort undergoing PCI (*n = *791)				
Female sex	10.30	4.50	1.80–11.27	0.001
Sheath size > 6F	8.88	4.37	1.66–11.52	0.003
Creatine kinase (per 10U/L)	6.66	1.02	1.01–1.04	0.01
Age (per year)	5.40	0.95	0.91–0.99	0.02
Body mass index (per 1 kg/cm^2^)	4.57	0.88	0.78–0.99	0.03

Variables sorted according to their strength of association with radial artery occlusion as determined by Wald chi-square. Maximum catheter size was excluded from analyses due to a strong interaction with sheath size as well as body weight due to a strong interaction with body mass index

*CI* confidence interval; *PCI* percutaneous coronary intervention

Regression analyses showed strong interactions of PCI during the index angiography with several other variables for prediction of radial artery occlusion. These variables include body mass index, smoking status, creatine kinase and most of periprocedural characteristics. Therefore, additional regression analyses were performed for patient with and without PCI. The univariate analyses are shown in Supplementary Tables A and B.

**Fig. 2:**
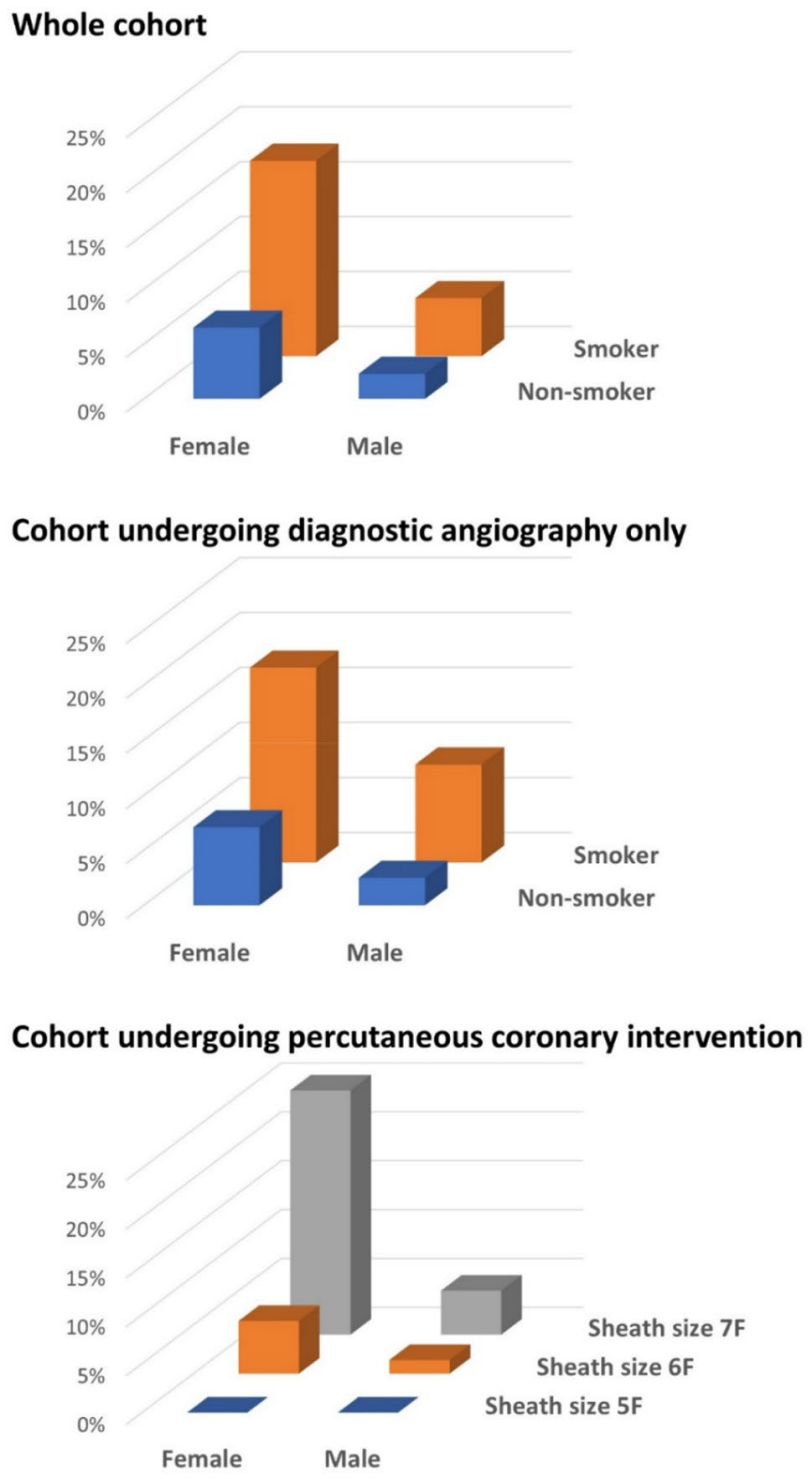
Incidence of radial artery occlusion in subgroups of key predictors

Multivariable binary logistic regression analyses identified active smoking as strongest independent predictor for radial artery occlusion in patients undergoing only diagnostic angiography followed by female sex and time of angiography. Active smokers had a more than three-fold increased risk for radial occlusion as compared to non-smokers (Fig. 2). In patients undergoing PCI, female sex was identified as the strongest independent predictor of radial occlusion followed by sheath size larger than 6 F, levels of creatine kinase, age, and body mass index. Female sex and use of larger sheaths were both associated with a more than fourfold increased risk for radial occlusion (Fig. 2).

### Antithrombotic treatment of radial artery occlusion

Patients diagnosed with a new radial artery occlusion received the recommendation for oral anticoagulation regardless of symptoms for at least 30 days according to the local standard of care. These patients were also scheduled for a clinical follow-up after 30 days. Of the 93 patients with radial artery occlusion, a complete follow-up including a repeat duplex sonography was available in 94% of patients (87 of 93). The reason for missing follow-up in the remaining six patients were non-cardiovascular death in two patients and patient refusal in 4 patients (mainly due to long distance travelling or limited mobility due to comorbidities). From the remaining 87 patients, 82% (71/87) received an oral anticoagulation. Key reasons for missing oral anticoagulation were planned major surgery within the next few days (mainly bypass and/or valve surgery) or an estimated high bleeding risk according to the treating physician (mainly history of recent major bleeding or high age).

The selection of oral anticoagulation was left to the treating physician. The majority of patients received NOACs (66 out of 71: rivaroxaban *n = *51, apixaban *n = *11, edoxaban *n = *6, dabigatran *n = *1) and only 5 patients the vitamin K antagonist phenprocoumon.

**Fig. 3:**
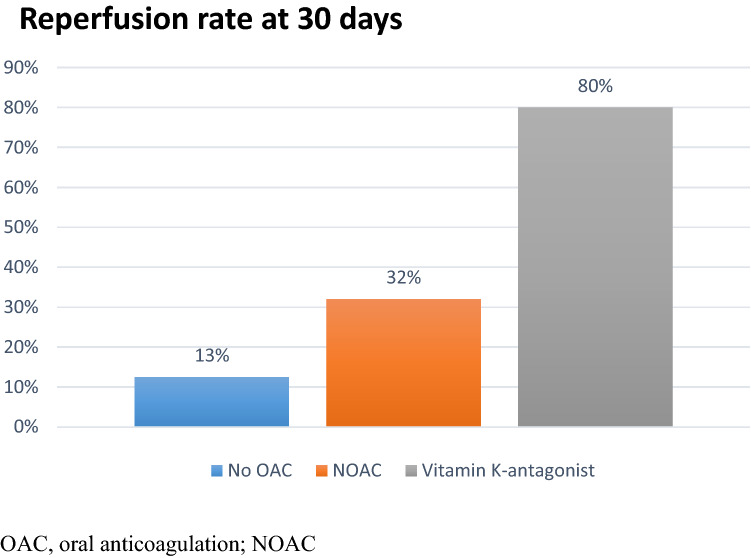
Reperfusion rates of radial artery at 30 days. *OAC* oral anticoagulation; *NOAC*, novel oral anticoagulant

The reperfusion rate of the radial artery at 30 days was 13% in patients receiving no oral anticoagulation and 32% in patients with oral anticoagulation. The highest reperfusion rate was seen in patients anticoagulated with phenprocoumon (4 out of 5; Fig. 3).

## Discussion

This study evaluated the incidence of vascular access site complications following transradial coronary angiography in a large cohort of unselected patients including a significant proportion of patients undergoing complex coronary intervention with large-bore access. Key finding was that the contemporary radial occlusion rate was with 4.6% lower as compared to many previously reported results [8, 11, 15]. Another key finding was that the incidence of other relevant complications was similarly low and, in particular, relevant peripheral neurologic or major bleeding even did not occur. These excellent results might be explained by the use of modern hydrophilic sheaths, improved puncture techniques or operator experience, and a standardized radial compression procedure.

Radial patency was evaluated on the day following coronary angiography. This early point of time was chosen since the majority of patients were discharged on this day. In previous studies, radial artery patency was evaluated from 24 h up of 30 days following the procedure [8, 13]. Even if direct comparison of different cohorts has major limitations, it appears if the incidence of radial occlusion might have been higher in studies with early testing as compared to studies with a later evaluation of this endpoint [10]. The overcome this potential limitation in the present study, patients diagnosed with radial artery occlusion were scheduled for a clinical follow-up demonstrating that the occlusion persisted in two of three patients despite intensified anticoagulation. This could indicate that rather traumatic than thrombotic factors might be the key mechanism leading to postprocedural radial occlusion.

In the present study, all patents were screened by duplex ultrasonography regardless of symptoms. This is an important feature of this study and might explain differences as compared to previous data. Earlier studies have infrequently reported even lower radial occlusion rates of less than 4%. However, these were mainly retrospective analyses with lower sample sizes. In some analyses, only symptomatic patients were apparently sent for ultrasound examination [16]. Even recent randomized trials demonstrating the superiority of the radial approach did only focus on clinical overt complications and did not screen for this complication [6]. As demonstrated in the present study, the majority of patients with radial occlusion were asymptomatic and patients with or without palpable pulse were found in both groups with or without radial occlusion. Thus, some asymptomatic patients with probably persistent occlusion might have been missed in previous studies. Apart from the routine ultrasound-based screening, further key features of the present study are the large sample size, which is one of the largest cohorts for this setting published so far, the consecutive enrollment of patients, which reduces the potential for selection bias, as well as the analysis of multiple variables that might impact on the incidence of radial occlusion. Given the enrollment in a tertiary care center, patients representing the whole spectrum of coronary diagnostics and interventions could be enrolled starting from the simple diagnostic angiography of young patients with planed valvular surgery to very complex and time-consuming coronary interventions needing large-bore access.

Overall, patients diagnosed with radial occlusion were more often female, had a lower prevalence of arterial hypertension and less frequently a history of coronary heart disease, which might be explained by their younger age. However, these patients were more often active smokers, demonstrated less frequently an obstructive coronary artery disease needing revascularization and had higher creatine kinase levels, which might point to a higher level of physical activity.

Young age has already previously been identified as predictor for radial artery occlusion [10, 11]. This might be explained by the higher sympathetic reactivity in younger individuals leading to a higher risk for vascular spasm as compared to elderly individuals with more atherosclerotic changes of the vascular wall and decreased sympathetic tone. A recent study assumed that higher age might be associated with ischemic pre-conditioning leading to larger artery diameter, based on the finding that patient’s age and body height was associated with a significant positive correlation with the radial artery diameter [15]. This finding is also in line with the present study demonstrating that low body height and low body weight were also predictors for radial occlusion. However, in multivariable models, neither body height nor body weight were independent predictors of radial occlusion.

Another analysis of 1706 patients undergoing transradial catheterization found a correlation of radial artery diameter with female gender, but not with body mass index [17]. This might explain why female sex emerged as strongest independent predictor for radial occlusion in almost all analyses of our cohort. It is well known that female patients trend to have smaller radial artery diameters as compared to male patients [11, 17]. The mismatch of vascular diameter and sheath size could have led to mechanical vascular damage. This potential pathomechanism is also underscored by the finding that the combination of female sex and use of a larger sheath size were associated with a more than four-fold increased risk for radial occlusion. Apart from mechanical vascular damage, vascular spasm might be one of the key mechanisms leading to radial vascular occlusion. Previous data demonstrated that female sex, younger age and lower body mass index are independent predictors of radial artery spasm [18]. Another study found only female sex as independent predictor for radial spasm, which was present in 10.3% of female patients [19]. However, procedural and fluoroscopy time showed also a trend to higher incidence of radial spasm in this analysis.

The last key predictor of radial occlusion in the present cohort was active smoking, which can be also associated with vascular spasm and has previously been reported to be associated with radial occlusion [20]. However, this association was in the present cohort only be seen for diagnostic angiography indicating that in patients undergoing coronary intervention, other variables such as sheath size might play a more important role.

Thus, pathogenesis of radial artery occlusion appears to be multifactorial. Sheath insertion and a mismatch between radial artery diameter and sheath size can lead to vascular damage and create a pro-thrombotic environment [21]. The evolution in techniques for radial access such as the use of hydrophilic sheaths might have reduced these effects given the lower incidence of radial occlusion in our cohorts as compared to previous reports. However, it is uncertain if these advances can also reduce the other key mechanism, which might be vascular spasm.

The results of the present analysis can certainly help to identify patients at risk for radial occlusion. Most cases of postprocedural radial occlusion occurred in patients undergoing only diagnostic coronary angiography (69 out of 93 in total) and in particular in female patients (*n = *43) with an incidence of 8.3%. Another key subgroup of patients with only diagnostic angiography were active smokers with an incidence of radial occlusion of 11.7% (*n = *23). Thus, one potential approach for risk reduction in these subgroups could be non-invasive imaging, which appears feasible in particular for younger patients with a lower prevalence of vascular calcifications. Current guidelines recommend coronary computed tomography angiography in selected cohorts due to the excellent outcomes demonstrated in patients presenting to the emergency department with low-to-intermediate pre-test probability for ACS. In addition, computed tomography imaging can exclude other causes of acute chest pain [22]. Other potential approaches for prevention of radial occlusion include a more careful vascular compression following angiography as well as the use of a far more distal vascular access [9, 23, 24].

In patients needing coronary intervention, key for reduction of radial occlusion appears to be the correct selection of sheath size. Overall, the use of large-bore sheaths was clinically safe in the present cohort with not any major bleeding or neurologic event, which is in line with previous clinical data [6]. However, similar as in other cohorts the incidence of radial occlusion increased with rising sheath size. In the Leipzig registry, an incidence of radial occlusion of 30.5% was seen if 6 F sheaths were used [8]. Another study using only 6 F sheaths demonstrated a radial artery diameter ≤ 2.5 mm and radial artery to sheath ratio < 1 as independent predictors for postprocedural occlusion of radial artery [11]. In the present study, the effect of rising sheath size was significantly dependent on sex with only a limited increase of incidence in male patients but a dramatic rise in female patients. Women receiving a 7 French sheath had an incidence of radial occlusion of 25%. Thus, aiming for smaller sheath sizes and probably pre-interventional sizing of the radial artery for sheath size selection might be potential approaches to reduce this vascular complication.

The optimal treatment for radial artery occlusion is still not well defined. Mechanical interventions such as an approach with transient ulnar compression has been suggested [12]. The most commonly described antithrombotic approach is the use of low-molecular-weight heparin for 7–30 days [25, 26]. The present study evaluated the use of oral anticoagulation, which represents the local standard of care. Even if the reperfusion rate appeared to be higher with oral anticoagulation as compared to no anticoagulation, the clinical success rate with oral anticoagulation of less than 1/3 was limited. This finding suggests that the local trauma might be more important than thrombotic mechanism. The missing association of antiplatelet or anticoagulatory treatment on admission with radial artery occlusion supports this theory. It is difficult to compare these results with previous data since in the majority of these studies, only symptomatic patients were treated. This might explain the higher success rates with low-molecular-weight heparin of about 87% [26].

### Limitations

This is a monocentric study with all adherent limitations. The majority of patients received a 6 F sheath and only about 10% of patients a 5 F or 7 F sheath limiting the informative value for these less frequently used sizes.

Radial occlusion was examined one day following coronary angiography given that most patients were discharged on this day. It cannot be excluded that evaluation of the primary endpoint at a later point of time would have shown different result. Data on treatment of radial occlusion were only observative and the majority of patients received oral anticoagulation, which was not standardized. Thus, this study cannot determine the natural course of radial occlusion or if other types of treatment would have had a higher success rate. Even if there was a standard for dosing of periinterventional drugs such as heparin or nitroglycerine, preferences of the operator or clinical circumstances have certainly caused some interindividual variations that might bias our results. Other potential reasons for bias are undetected differences in operator experience or radial vasomotility or spasm.

## Conclusion

The incidence of radial artery occlusion following coronary angiography in contemporary practice is with less than 5% lower as compared to previous studies. Female sex and smoking status are the strongest independent predictors of radial artery occlusion followed by procedural variables. These results might guide clinical decision making by facilitating identification of patients at risk for postprocedural radial occlusion, which might be preferable candidates for non-invasive imaging or use of smaller sheath sizes. The limited effectiveness of oral anticoagulation for treatment of radial artery occlusion suggests a primarily traumatic than thrombotic mechanism of this complication.

## Supplementary Information

Below is the link to the electronic supplementary material.

Supplementary file1 (DOCX 33 kb)

## Abbreviations

BARC: Bleeding academic research consortium
CABG: Coronary artery bypass grafting
CI: Confidence interval
CTO: Chronical total occlusion
EF: Ejection fraction
LV: Left ventricle
PCI: Percutaneous coronary intervention
TIA: Transient ischemic attack
OAC: Oral anticoagulant
NOAC: Novel oral anticoagulant
