# Supplementary material for: Incidence and predictors of radial artery occlusion following transradial coronary angiography: the proRadial trial
Source: Clin Res Cardiol. 2022 Sep 8;112(9):1175–85. doi: 10.1007/s00392-022-02094-z (PMC10449957; doi:10.1007/s00392-022-02094-z)
Supplement: Supplementary file 1 — Supplementary file1 (DOCX 33 kb) [file 392_2022_2094_MOESM1_ESM.docx]

**Supplemental materials for**

**Incidence and predictors of radial artery occlusion following transradial coronary angiography: the proRadial trial**

Julia Schlosser^1^, Laura Herrmann^1^, Tanja Böhme^1^, Karlheinz Bürgelin^1^, Nikolaus Löffelhardt^1^, Thomas Nührenberg^1^, Kambis Mashayekhi^1^, Christian M. Valina^1^, Franz-Josef Neumann^1^, Willibald Hochholzer^2^

^1^University Heart Center Freiburg · Bad Krozingen, Department of Cardiology and Angiology II, Campus Bad Krozingen, Germany

^2^Department of Internal Medicine and Cardiology, Klinikum Wuerzburg Mitte, Würzburg, Germany

**Address for correspondence**

Julia Schlosser, MD

University Heart Centre Freiburg · Bad Krozingen

Department of Cardiology and Angiology II

Suedring 15, 79189 Bad Krozingen, Germany

Phone: +49-7633-402-0 / Fax: +49-7633-402-2489

Email: [Julia.Schlosser@uniklinik-freiburg.de](mailto:Julia.Schlosser@uniklinik-freiburg.de)

ORCHID-ID: 0000-0001-6025-6137

**Supplementary Table A:** Univariable binary logistic regression analyses for radial occlusion in patients undergoing only diagnostic angiography.

| **Baseline characteristics** | **Odds Ratio** | **95%-CI** | **P-value** |
| --- | --- | --- | --- |
| Age (per year) | 0.98 | 0.96-1.00 | 0.08 |
| Female sex | 2.39 | 1.45-3.94 | 0.001 |
| Body height (per 1cm) | 0.97 | 0.94-0.99 | 0.03 |
| Body weight (per 1kg) | 0.98 | 0.97-1.00 | 0.13 |
| Body mass index (per 1kg/cm²) | 0.99 | 0.94-1.04 | 0.69 |
| Body-surface area (per 1m²) | 0.29 | 0.09-0.92 | 0.04 |
| Impaired LV function (EF < 55%) | 0.93 | 0.51-1.67 | 0.80 |
| Coronary artery disease | 0.76 | 0.46-1.24 | 0.27 |
| Congestive heart failure | 1.52 | 0.90-2.59 | 0.12 |
| Previous transradial angiography | 0.92 | 0.53-1.62 | 0.78 |
| Previous CABG | 0.57 | 0.08-4.21 | 0.58 |
| Arterial hypertension | 0.69 | 0.41-1.16 | 0.16 |
| Active smoking | 2.78 | 1.65-4.72 | <0.001 |
| Diabetes | 1.01 | 0.56-1.83 | 0.96 |
| Oral anticoagulant | 0.74 | 0.37-1.47 | 0.39 |
| β-Blockers | 0.83 | 0.51-1.35 | 0.45 |
| Nitrates | 1.88 | 0.56-6.36 | 0.31 |
| Calcium channel blockers | 0.77 | 0.41-1.47 | 0.44 |
| High sensitivity troponin T (per 1ng/L) | <0.01 | <0.01-373 | 0.23 |
| Creatine kinase (per 10U/L) | 0.77 | 0.34-1.77 | 0.55 |
| C-reactive protein (per 1mg/L) | 1.02 | 0.75-1.22 | 0.81 |
| Haemoglobin (per 1g/dL) | 0.97 | 0.87-1.07 | 0.55 |
| Thrombocytes (per 100x10³/µL) | 1.34 | 1.00-1.79 | 0.049 |
| White blood cells (per 1000/µL) | 1.05 | 0.99-1.11 | 0.10 |
| **Peri- and postprocedural characteristics** | |  |  |
| Multiple puncture attempts | 1.77 | 0.40-7.75 | 0.45 |
| Time of examination (per min) | 1.01 | 0.99-1.02 | 0.07 |
| Sheath size >5F | 1.26 | 0.39-4.12 | 0.70 |
| Maximum catheter size >5F | 0.79 | 0.49-1.29 | 0.35 |
| Number of catheter exchanges | 0.97 | 0.80-1.17 | 0.71 |
|  |  |  |  |

CI, confidence interval; LV, left ventricle; EF, ejection fraction; PCI, percutaneous coronary intervention; CABG, coronary artery bypass grafting. Missing results for variables with no events in a subgroup of analysis.

**Supplementary Table B:** Univariable binary logistic regression analyses for radial occlusion in patients undergoing PCI.

|  | **Odds Ratio** | **95%-CI** | **P-value** |
| --- | --- | --- | --- |
| **Baseline characteristics** |  |  |  |
| Age (per year) | 0.96 | 0.93-1.00 | 0.06 |
| Female sex | 3.59 | 1.59-8.14 | 0.002 |
| Body height (per 1cm) | 0.97 | 0.93-1.02 | 0.27 |
| Body weight (per 1kg) | 0.97 | 0.94-1.00 | 0.05 |
| Body mass index (per 1kg/cm²) | 0.91 | 0.81-1.01 | 0.08 |
| Body-surface area (per 1m²) | 0.13 | 0.02-1.09 | 0.06 |
| Impaired LV function (EF < 55%) | 0.25 | 0.06-1.06 | 0.06 |
| Congestive heart failure | 0.51 | 0.07-3.86 | 0.52 |
| Previous transradial angiography | 1.25 | 0.56-2.84 | 0.59 |
| Arterial hypertension | 0.50 | 0.20-1.23 | 0.13 |
| Active smoking | 1.62 | 0.63-4.16 | 0.32 |
| Diabetes | 1.02 | 0.43-2.42 | 0.96 |
| Oral anticoagulant | 0.60 | 0.18-2.03 | 0.41 |
| β-Blockers | 0.50 | 0.22-1.14 | 0.10 |
| Nitrates | 3.58 | 0.79-16.32 | 0.10 |
| Calcium channel blockers | 1.09 | 0.43-2.78 | 0.86 |
| High sensitivity troponin T (per 1ng/L) | 1.67 | 0.93-3.00 | 0.09 |
| Creatine kinase (per 10U/L) | 1.02 | 1.01-1.04 | 0.007 |
| C-reactive protein (per 1mg/L) | 0.82 | 0.44-1.55 | 0.55 |
| Haemoglobin (per 1g/dL) | 0.87 | 0.72-1.06 | 0.17 |
| Thrombocytes (per 100x10³/µL) | 1.08 | 0.71-1.64 | 0.72 |
| White blood cells (per 1000/µL) | 0.96 | 0.80-1.15 | 0.66 |
|  |  |  |  |
| **Peri- and postprocedural characteristics** | |  |  |
| Acute myocardial infarction | 0.89 | 0.26-3.04 | 0.85 |
| Multiple puncture attempts | 4.56 | 0.98-21.16 | 0.05 |
| Time of examination (per min) | 1.01 | 0.99-1.01 | 0.17 |
| Sheath size >6F | 2.81 | 1.17-6.71 | 0.02 |
| Maximum catheter size >6F | 2.49 | 1.01-6.13 | 0.048 |
| Number of catheter exchanges | 0.87 | 0.64-1.18 | 0.37 |
|  |  |  |  |

CI, confidence interval; LV, left ventricle; EF, ejection fraction; PCI, percutaneous coronary intervention; CABG, coronary artery bypass grafting. Missing results for variables with no events in a subgroup of analysis.
